# Intermittently “Pre-Excited” ECG after Accessory Pathway Ablation: Unsuccessful Procedure or a Complication?

**DOI:** 10.1155/2012/289067

**Published:** 2012-08-07

**Authors:** Evgeny Mikhaylov, Elnara Voitkovskaya, Dmitry Lebedev

**Affiliations:** Department of Electrophysiology and Cardiac Pacing, Almazov Federal Heart, Blood and Endocrinology Centre, 197341 Saint Petersburg, Russia

## Abstract

A 52-year-old woman with previously unsuccessful posteroseptal pathway ablation using radiofrequency energy presented with intermittently occurring short PR followed by a wide QRS complex, and complaining of palpitations with characteristics different from previous history. During a second electrophysiological procedure no signs of preexcitation were found. Ventricular discharges with fusion with sinus beats were revealed, and catheter ablation of premature contractions originating from the proximity to previous accessory pathway was carried out.

## 1. Introduction

**Figure 1:**
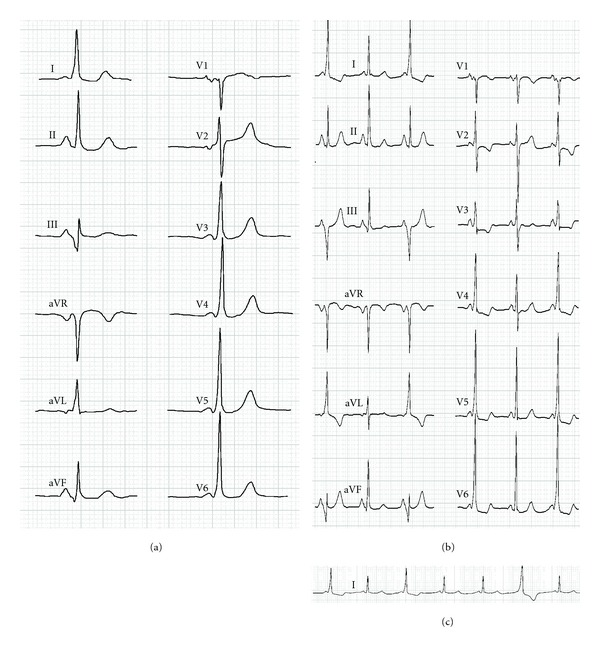
(a) Surface ECGs of the patient. (a) The ECG before the first ablation. A short PR interval, a delta wave. and a widened QRS are clearly seen. Panels (b) and (c) show ECG pattern after the first ablation. (b) Twelve ECG leads show three beats: the first and the third are with the short PR and the wide QRS, while the second beat is normal. (c) The continuous recording of the lead I shows alternation of the PR interval and the QRS.

A 52-year-old woman suffering from palpitations was referred to the electrophysiology laboratory. Her rest ECG showed intermittent shortening of a PR interval followed by a wide QRS. The patient had previous radiofrequency (RF) modification of a posteroseptal accessory pathway (AP) one month ago (Figure 1(a)).

## 2. Case Presentation

During the previous procedure the AP with ante- and retrograde conduction was diagnosed and ablated via the coronary sinus (CS) at the ostium of the middle cardiac vein. RF ablation was carried out using an open-irrigated 3.5 mm tip ablation catheter (30 W, 42°C). The ablation resulted in disappearance of retrograde conduction over the AP (as was shown by parahisian pacing) and a narrow QRS during the sinus rhythm; however fast (330 ms) and programmed atrial pacing showed preexcited QRS with a very long stimulus-R interval, and with prolonged AP-effective refractory period (ERP) (280 ms). Subsequent mappings and ablations from right and left chambers had no further effect. Since the AP was modified, and the reciprocating tachycardia was no more inducible, the procedure was stopped and considered as unsuccessful ablation. Five days later the patient complained of recurrent palpitations with “warm-up” and “cool-down” characteristics. The rest ECG showed sinus rhythm with intermittently appearing short PR intervals intervals being followed by wide QRS complexes suggestive of preexcitation (Figures 1(b) and 1(c)). The QRS complexes with the short PR interval were even wider than before the first AP ablation, and a slight difference in QRS morphology (leads III and aVF) was noted. No tachycardia was documented. The patient was referred for a second electrophysiological procedure.

## 3. Electrophysiological Study and ****Catheter Ablation

During the second procedure, a decapolar diagnostic catheter (Webster, Biosense Webster, Diamond Bar, CA, USA) was inserted into the CS; a quadripolar diagnostic catheter (Biosense Webster) was placed into the His position, and an open-irrigated ablation catheter (Biosense Webster) was used for diagnostic maneuvers and ablation.

**Figure 2:**
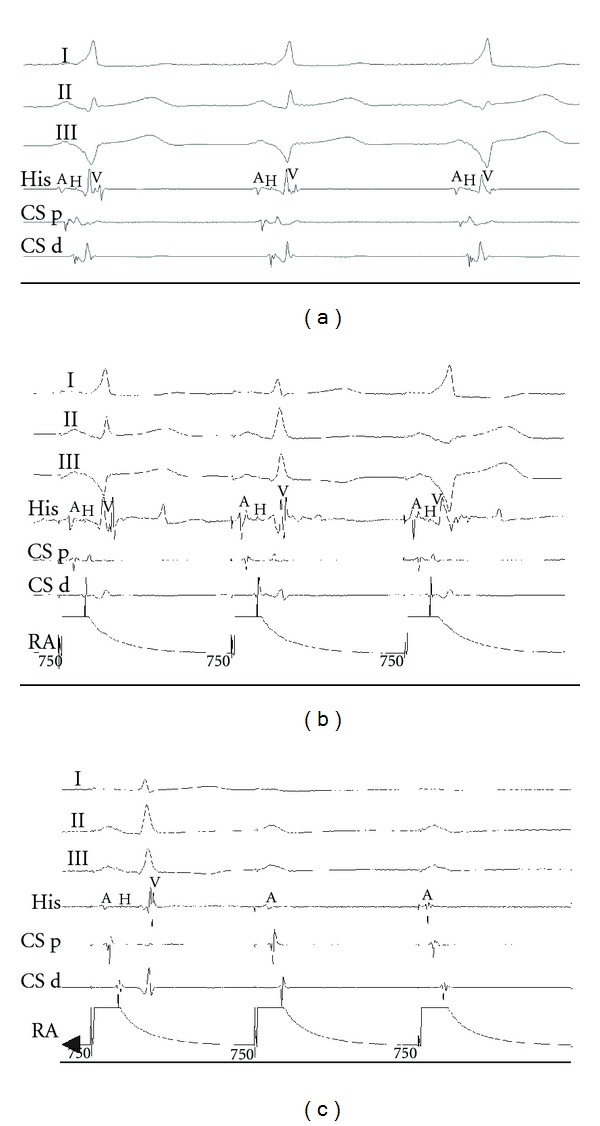
Surface ECG and intracardiac electrograms during the second electrophysiologic testing. (a) During sinus rhythm. A short PR interval followed by a wide QRS is clearly seen. Note short HV interval on the His electrogram. (b) During right atrial pacing. The first and third beats are with the short PR and the wide QRS, and second paced beat is a normal sinus beat. (c) After adenosine bolus. Note transient complete AV block and no QRS widening. His, His electrogram; CSp, electrogram from the proximal pairs of the coronary sinus catheter; CSm, from middle pairs; CSd, from distal pairs; RA, electrograms from a catheter located in high right atrial position.

 At the baseline, sinus rhythm with the short PR and the wide QRS was present (Figure 2(a)). Right ventricle pacing showed decremental conduction. Parahisian pacing showed no signs of conduction over the AP. During right atrial pacing with rate just above spontaneous sinus rate, alternation of the PR interval and QRS morphology change (with shortening of the HV interval) was present (Figure 2(b)). Faster pacing resulted in a normal QRS and decremental AH prolongation. Programmed atrial pacing at different basic cycle lengths showed a narrow QRS with decremental atrioventricular (AV) conduction. Adenosine bolus during right atrial asynchronous pacing resulted in transient AV conduction block with no QRS widening (Figure 2(c)). No tachycardia was inducible.

The two following mechanisms can be proposed for the observed phenomena. The RF ablation during the first procedure could significantly change conduction properties of the AP, resulting in decremental and/or transient conduction [1]. Narrow QRS during atrial pacing could be due to prolonged antegrade ERP of the AP being longer than that of the AV node. Programmed pacing and adenosine administration may coincide with the development of spontaneous conduction block in the AP and, therefore, reveal no preexcitation. Other mechanism could be related to ventricular focal discharges with rate close to the sinus rate resulting in appearance of fusion QRS beats, therefore, mimicking recovered preexcitation.

**Figure 3:**
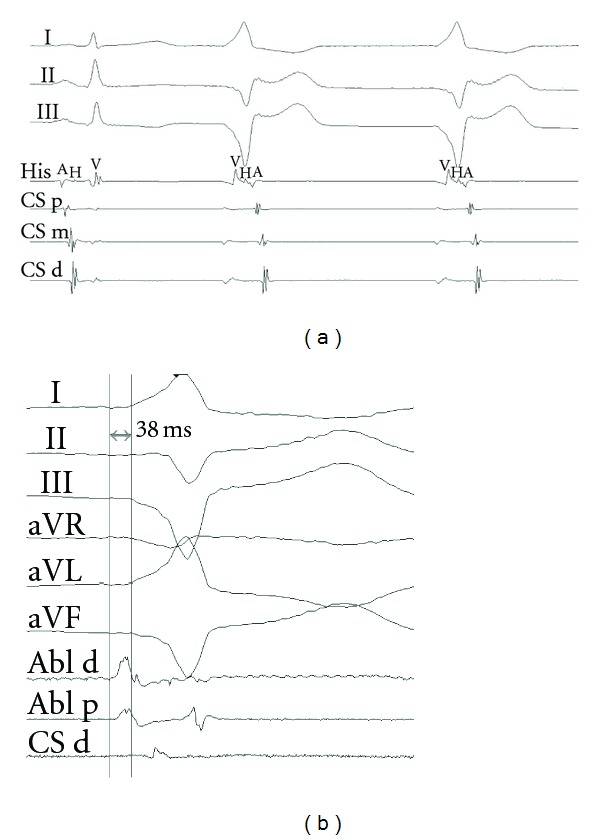
(a) The ventricular discharges with retrograde conduction over the AV node (second and third beats). (b) Early presystolic activity at the ablation site in the middle cardiac vein. Abl d, electrogram from the distal pair of the ablation catheter; Abl p, from the proximal pair.

To uncover a possible coherent ventricular rhythm a maneuver to slow sinus rhythm was required. It was known that the patient had propofol-induced bradycardia in the past [2]. A bolus of propofol was administered, and the sinus rhythm decreased from 72 to 58 bpm. A dissociated ventricular rhythm with the same morphology and with retrograde ventriculo-atrial conduction over the AV node was uncovered (Figure 3(a)). Atrial pacing showed prolonged antegrade ERP of the AV node. Right ventricle pacing resulted in decremental conduction over the AV node, as proved by parahisian pacing. Activation mapping of the ventricular ectopy showed the most early activation site in the middle cardiac vein, slightly deeper in comparison with the point of previously ablated AP (Figure 3(b)). Ablation at this point immediately terminated the ectopic activity. Electrophysiologic testing showed no signs of preexcitation. Subsequent 3-month followup, ECGs and Holter monitoring were uneventful.

## 4. Discussion

It has been shown that the extent of tissue injury produced by RF ablation in vivo appears to be larger than the region of acute coagulation necrosis, which result in a border zone of acutely injured but viable myocardium. A secondary inflammatory response and/or ischemia as a consequence of microvascular damage may cause progression of tissue injury within the border zone. [3]. This case represents two phenomena (delayed effect of RF ablation and spontaneous ventricular activity from the vicinity of previous ablation site) occurring due to a unique feature of the RF ablation expanding of necrosis and inflammation within the border zone of the lesion. Although delayed effect of AP ablation and focal activity from a previously ablated area have been previously published [4, 5], both phenomena in one patient have not previously been reported.

We believe that the ventricular spontaneous activity did not originate from the remnant AP since extensive mapping did not show any potential preceding the activation at the ablated point. Presence of bidirectional block within the accessory pathway, together with overdrive suppression with either atrial or ventricular pacing, suggests a slow ventricular discharges rather than automaticity of the accessory pathway. Most probably, the ectopic rhythm originated from transition zone around the RF lesion. Different fusion degree of the spontaneous ventricular activity and normal conducted beats produced alternation of QRS morphology (Figures 1(c), 2(a) and 2(b)). The identity of the ectopic rate and atrial rate (either sinus and paced) resulted in misdiagnosis of recovered conduction over the AP. Pacing at rates significantly higher than spontaneous sinus rate resulted in suppression of the ectopic activity.

Since no palpitations have been reported after the second procedure, occasional acceleration of the ventricular discharges could be suggested as a probable mechanism of a tachycardia occurring after the initial ablation.

This case has an important implication; careful ECG examination using noninvasive tests (e.g., maneuvers to slow sinus rhythm) should be considered before making the diagnosis of recovered AP conduction, even after “unsuccessful” ablation.

## Abbreviation

AP: Accessory pathway
AV: Atrioventricular
CS: Coronary sinus
ECG: Electrocardiogram
ERP: Effective refractory period
RF: Radiofrequency.
